# Caffeine and sodium bicarbonate supplementation alone or together improve karate performance

**DOI:** 10.1186/s12970-019-0313-8

**Published:** 2019-10-17

**Authors:** Sajjad Rezaei, Kazem Akbari, Daniel E. Gahreman, Amir Sarshin, Montassar Tabben, Mojtaba Kaviani, Alireza Sadeghinikoo, Majid S. Koozehchian, Alireza Naderi

**Affiliations:** 1 Physical Education and Sport Sciences Department, Faculty of Humanities, Tarbiat Modares University, Tehran, Iran; 2 Department of exercise physiology, Faculty of Physical education and sport sciences, Kharazmi University, Tehran, Iran; 3 Raad Sports Club, Tehran, Iran; 4 College of Health and Human Sciences, Charles Darwin University, Darwin, Australia; 5 Faculty of Physical education and sport sciences, Department of Exercise Physiology, Karaj Branch, Islamic Azad University, Karaj, Iran; 6 Aspetar, Qatar Orthopaedic and Sports Medicine Hospital, Doha, Qatar; 7 School of Nutrition and Dietetics, Acadia University, Wolfville, Nova Scotia Canada; 8 Sport Medicine Center, Oxygen Sport Group, Tehran, Iran; 9 Department of Kinesiology, Jacksonville State University, Jacksonville, AL 36265 USA; 10 Sports Physiology Department, Islamic Azad University, Boroujerd Branch, Boroujerd, Iran

**Keywords:** Karate, Ergogenic aid, Caffeine, Sodium bicarbonate, Blood lactate, Time to exhaustion, Karate-specific aerobic test, Rate of perceived exertion

## Abstract

**Background:**

The ergogenic properties of acute caffeine (CAF) and sodium bicarbonate (NaHCO_3_) ingestion on athletic performance have been previously investigated. However, each sport has unique physiological and technical characteristics which warrants optimizing supplementations strategies for maximizing performance. This study examined the effects of CAF and NaHCO_3_ ingestion on physiological responses and rate of perceived exertion during a Karate-specific aerobic test (KSAT) in competitive karatekas.

**Methods:**

In a double-blind, crossover, randomized placebo-controlled trial, eight Karatekas underwent five experimental conditions including control (CON), placebo (PLA), CAF, NaHCO_3_, and CAF + NaHCO_3_ before completing KSAT. Capsules containing 6 mg/kg BW CAF were consumed 50 min prior to a KSAT whilst 0.3 g/kg BW NaHCO_3_ was consumed for 3 days leading to and 120, 90, and 60 min prior to a KSAT. Time to exhaustion (TTE), rate of perceived exertion (RPE), and blood lactate (BL) were measured before, immediately after and 3 min following KSAT.

**Results:**

TTE was significantly greater following CAF, NaHCO_3_, and CAF + NaHCO_3_ consumption compared to PLA and CON. However, the differences between CAF, NaHCO_3_, and CAF + NaHCO_3_ were not statistically significant (*p* > 0.05). BL increased significantly from baseline to immediately after and 3 min following KSAT in all conditions (*p* < 0.01), while RPE at the end of KSAT was not significantly different between conditions (*p* = 0.11).

**Conclusions:**

Karate practitioners may benefit from the ergogenic effects of CAF and NaHCO_3_ when consumed separately or together.

## Introduction

Karate is a Japanese military martial art that has evolved into a modern combat sport and includes Kumite (non-contact fighting) and Kata (demonstration) [1]. Kumite is a 3-min sparring, and despite technical differences between karatekas, it consists of periods of technical movements interspersed by rhythmic bouncing at a lower intensity [2]. In Kumite, the oxidative phosphorylation is the predominant energy system during low-intensity bouncing (77.8 ± 5.8%), while ATP-PCr (4 ± 4.6%) and glycolysis (6.2 ± 2.4%) energy systems supply the energy for bursts of activities in attack and defence [2].

The by-products of the anaerobic glycolysis, namely H^+^ have detrimental effects on athletic performance; hence, reduction or neutralization of these byproducts may improve performance and time to exhaustion [3]. An indicator of glycolytic metabolism is the concentration of blood lactate (BL), which has been shown to increase in simulated and real Kumite competitions [4, 5]. Besides, it is reported that when H^+^ accumulation exceeds lactate formation, performance may be impaired by inhibiting key enzymes of the glycolytic pathway and impeding PCr resynthesis [6–8].

Furthermore, intracellular acidosis reduces the maximal shortening velocity, Ca^2+^ sensitivity, and extends the relaxation period. Consequently, the force and the rate of force development reduces which diminishes performance [9, 10]. Thus, maintaining an acid-base balance is a challenge during Kumite, when a large quantity of H^+^ ions is produced by the anaerobic energy pathway. Most exercise-induced H^+^ ions are immediately transported out of myofibers and buffered by circulating buffers namely bicarbonate [11, 12]. Sodium bicarbonate (NaHCO_3_) is an essential extracellular buffer that neutralizes the H^+^ ions to maintain blood pH levels [3, 13]. A recent study showed that ingestion of NaHCO_3_ prior to a high-intensity activity, enhanced the contribution of glycolytic metabolism and improved performance during simulated Taekwondo [14]. It is believed that the ingestion of NaHCO_3_ improves performance by reducing exercise-induced acidosis and delaying fatigue [15, 16]. Yet, sustained performance in competition is usually influenced by multiple factors including low ATP content, muscle acidosis, increased extracellular potassium, low muscle glycogen content, inadequate muscle PCr stores, and the central nervous system (CNS) fatigue [15, 17].

Caffeine (CAF), as a stimulant, affects the CNS via acting on adenosine receptors reducing fatigue and decreasing the rate of perceived exertion (RPE) [18–20]. It has been shown that acute CAF supplementation (5 mg/kg BW) in young female Karatekas led to a reduction of RPE and pain perception during 60% 1RM leg press [21]. Similarly, the ingestion of CAF improved reaction time and delayed fatigue during successive Taekwondo combats [22]. Furthermore, CAF could increase the glycolytic contribution to energy metabolism during simulated and real combat sports [23]; this increase in combat time and energy metabolism could lead to greater catecholamine release [24].

Theoretically, the synergic effects of CAF and NaHCO_3_ co-ingestion improve performance in combat sports by delaying CNS fatigue, reducing extracellular potassium accumulation, and elevating the extracellular buffering capacity [15]. In line, Judo performance was enhanced by acute co-ingestion of NaHCO_3_ (0.3 g/kg BW) and CAF (6 mg/kg BW), while ingestion of these supplements separately did not improve exercise performance [25]. Despite similarities between combat sports, there are considerable differences between the technical characteristics of Judo and Karate. For example, Judo consists of grappling and throwing techniques which require strength-endurance and power [26], while Karate is a striking sport which is characterized by a high speed attack and defense movements [27]. Also, it has been reported that the effort to pause ratio during Karate world cup competition was approximately 1:1–2 [1] whereas this ratio is 2:1 or 3:1 in Judo competitions [26]. Such differences limit the generalization of findings in previous research in Judo to sports such as Karate. Therefore, the current study aimed to investigate the acute effects of CAF and four day ingestion of NaHCO_3_ either together or separately on time to exhaustion (TTE), RPE, and BL during a Karate-specific aerobic test (KSAT). We hypothesized that co-ingestion of CAF and NaHCO_3_ improve Karate performance greater than ingestion of these supplements separately.

## Methods

### Participants

All members of the Raad Karate club were invited to participate in this study. The Raad Karate club was ranked 4th in 2018 Iranian first division Karate league and had largest number of competitive Karatekas. This study was conducted in one Karate club to eliminate the potential effects of variations in training programs between different clubs. Karatekas were included in this study if they had more than 5 years experience in Karate, did not consume any supplements 3 months before and during the study, and were not heavy CAF users (CAF ≤ 125 mg/d). Altogether, 10 Karatekas participated in the study. However, two participants were invited to Iran’s national Karate camp and underwent a different training plan; therefore, they could not participate in this study anymore. A total of eight Karatekas (age: 20.5 ± 2.4 y; height: 1.78 ± 0.06 m; body mass: 67.8 ± 7.7 kg; body fat percentage, 10 ± 3) completed the study. To determine whether the number of participants were adequate for this study, we used a priori power analysis using the G*Power 3.1.9.2 [28]. To obtain a statistical power at 0.9 level using repeated measure ANOVA, six participants were required to detect a moderate effect of conditions on time to exhaustion as the main dependent variable of the study.

The study was carried out during a 6-week preparation phase of the annual training program. During the transition phase, Karatekas trained six sessions per week, including three Karate specific training sessions and three conditioning sessions, including strength training and Karate-specific fitness.

Height and body mass of participants were measured in the first visit using an electronic stadiometer SECA 217 (Seca Ltd., Hamburg, Germany) and a calibrated Seca 770-floor digital scale (Seca Ltd., Hamburg, Germany), respectively. Body composition was determined using a bioelectrical impedance analysis (InBody 270 Biospace, Seoul, Korea).

This study was approved by the human ethics research committee of the Sport Sciences Research Institute of Iran (Code: IR.SSRI.REC.1397.216).

### Experimental design

The present study adopted a double-blind, crossover, randomized, placebo-controlled design. All supplements were prepared and administered by an independent pharmacist to ensure both researchers and participants were blind to conditions. After completing two KSAT familiarization sessions, participants were assigned into five conditions including: CAF, NaHCO_3_, a combination of CAF and NaHCO_3_, placebo (PLA), and control (CON). The order of these conditions was selected randomly for each participant to control for the potential effects of training variables during the study period. The results of the control (CON) session were used as a baseline for comparative analyses. The washout period was 7 days and to account for circadian variations, all assessments were conducted at the same time (between 09:00 AM and 12:30 PM) and the same day each week.

### Karate specific aerobic test

Participants performed a 10-min warm-up routine consisted of a 7-min dynamic stretching and low-intensity jogging followed by a 3-min specific warm-up including punches and kicks of a heavy bag. After a 2-min passive recovery, Karatekas performed a KSAT on a tatami, following the protocol proposed by Tabben et al. [29]. The reliability, validity, and procedure of the KSAT have been previously described in details [29]. Briefly, the test involved two attack combinations on a punching bag. Attack 1: a leading straight punch followed by a rear straight punch (kisamigyaku-zuki), and Attack 2: a rear leg roundhouse kick (mawashi-geri-chudan). The distance between Karatekas’ front foot and the punching bag was 1.5 m, allowing participants to complete each combination in 3 seconds. The test was progressive; whilst the time for completing the attack movements remained constant (3 s), recovery time between movements decreased until Karatekas reached exhaustion. During the recovery time, Karatekas performed rhythmic bouncing on their preferred guard similar to the real Kumite. Two auditory signals were used during the KSAT: the first signal indicated an attack and the second signal was for the rest period. The research team encouraged participants verbally to ensure the maximum power was delivered in each punch and kick. The test was terminated when Karatekas failed to complete two combinations and/or when Karatekas failed to execute correct forms of punches and kicks. The correct execution of techniques were subjectively assessed by two qualified coaches.

### Supplementation protocol

The supplements and PLA (cellulose) were packed in identical gelatin capsules (Iran Gelatin Capsule Co. Iran), and participants could not identify the capsules’ content. Supplementation started 3 days before each KSAT session with either NaHCO_3_ (0.3 g/kg BW/d, AGC Industries Co., China) or PLA which were consumed with breakfast, lunch, and dinner. This loading strategy was adopted as it has been shown to reduce the gastrointestinal (GI) discomfort and sustain the blood carbonate levels 1 day after NaHCO_3_ ingestion [30]. On the assessment day, capsules containing either NaHCO_3_ (0.1 g/kg BW) or PLA were consumed 120, 90, and 60 min before KSAT. A capsule containing either CAF 6 g/kg BW (Caffeine Anhydrous, CSPC Innovation Pharmaceutical Co., China) or PLA was consumed 50 min before KSAT, as it has been shown that the concentration of caffeine in blood peaks 30–60 min after consumption [31].

### Dietary control

Participants were instructed to avoid consuming any drinks and foods that contained baking soda, CAF, or alcohol throughout the study, and/or performing high-intensity exercise within 24 h before KSAT. A list of common food and beverages that were safe to consume and those to avoid was provided to participants. Although participants did not record dietary consumption throughout the study, they were advised to record their dietary intake 24 h prior to the first condition to replicate it before remaining sessions. To minimize potential gastrointestinal distress, participants consumed a standardized snack (white bread and boiled eggs) containing 1.5 g/kg BW carbohydrates, 20 g of protein, and 10 g fat 150 min before each KSAT.

### Blood lactate analysis

Blood samples were collected from karatekas’ earlobe before warm-up, immediately after, and 3 min following KSAT. The BL concentration (mmol/L) was measured by the photometric method, using a portable analyzer (Lactate Scout^+^ analyzer, SensLab GmbH, Germany).

### Heart rate and RPE measurement

The heart rate during KSAT was recorded with a Polar heart rate monitor (Polar, V800, H7 heart rate sensor, Electro, Oy, Kempele, Finland). In addition, the rate of perceived exertion (RPE) was recorded on the scale of 1–10 after each level of KSAT.

### Gastrointestinal questionnaire

A gastrointestinal questionnaire was used to assess the symptoms of gastrointestinal discomfort [32]. Participants selected values ranging from 0 to 9, where 0 indicated ‘no problem at all,’ and 9 indicated ‘the worst it has ever been’. The symptoms were considered severe when the score was equal to or greater than 5.

### Monitoring fatigue and training status

To minimize the effect of training volume and avoid overreaching, coaches were asked to maintain training volume and intensity throughout the study. The well-being Hooper index questionnaire [33] was used before each KSAT to monitor and assess the recovery and accumulated fatigue.

Moreover, the recovery among KSAT sessions was assessed using countermovement jumps (CMJ) [34]. The CMJ was performed three times using the procedures recommended by Maulder and Cronin (2005), and the highest jump was recorded for further analysis [35].

### Statistical analysis

Data were analyzed with SPSS 25.0 (SPSS Inc., Chicago, IL) and were presented in mean and standard deviation (SD). A One-Way repeated measure analysis of variance (ANOVA) was used to compare the effect of different supplementation on time to exhaustion (TTE), heart rate (HR), and RPE after each KSAT. The effects of different supplements and KSAT on BL were analyzed using a split-plot ANOVA. When the results revealed a significant difference between conditions, a Bonferroni post-hoc analysis was conducted to identify the differences. The effect size (ES) for simple effects was also calculated to verify the magnitude of the effect of each supplement on performance, values of 0.2, 0.6, 1.2, 2.0, 4.0 and > 4.0 were considered trivial, small, moderate, large, very large and extremely large, respectively.

## Results

### TTE, HR, and RPE

**Fig. 1:**
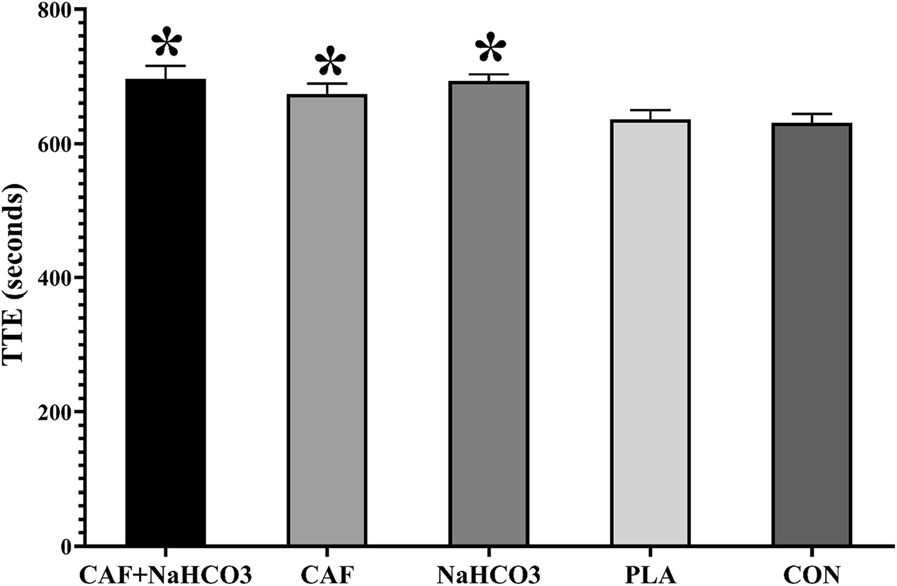
Time to exhaustion during karate-specific aerobic test in each treatment. TTE, time to exhaustion; CAF, caffeine; NaHCO3, sodium bicarbonate; PLA, placebo; CON, control. * Significant difference with PLA and CON (*P* < 0.05)

The results showed a significant effect of supplementation on TTE in karatekas during KSAT (Fig. 1.), *F* [4, 28] = 16.49, *p* < 0.001, ${\eta}_P^2$ = 0.70. Pairwise comparison revealed that TTE was significantly greater in CAF (674 ± 44 s, *p* = 0.001, ES = 0.89), NaHCO_3_ (693 ± 28 s, *p* = 0.015, ES = 1.69), and CAF + NaHCO_3_ (696 ± 56 s, *p* = 0.012, ES = 1.23) compared to PLA (636 ± 39 s). A significant difference was also observed in TTE between CAF (674 ± 44 s, *p* = 0.018, ES = 0.46), NaHCO_3_ (693 ± 28 s, *p* = 0.003, ES = 0.68), and CAF + NaHCO_3_ (696 ± 56 s, *p* = 0.011, ES = 0.56) compared to CON (631 ± 38 s). However, TTE differences between CAF, NaHCO_3_, and CAF + NaHCO_3_, and differences between CON and PLA were not statistically significant (*p* > 0.05).

**Fig. 2:**
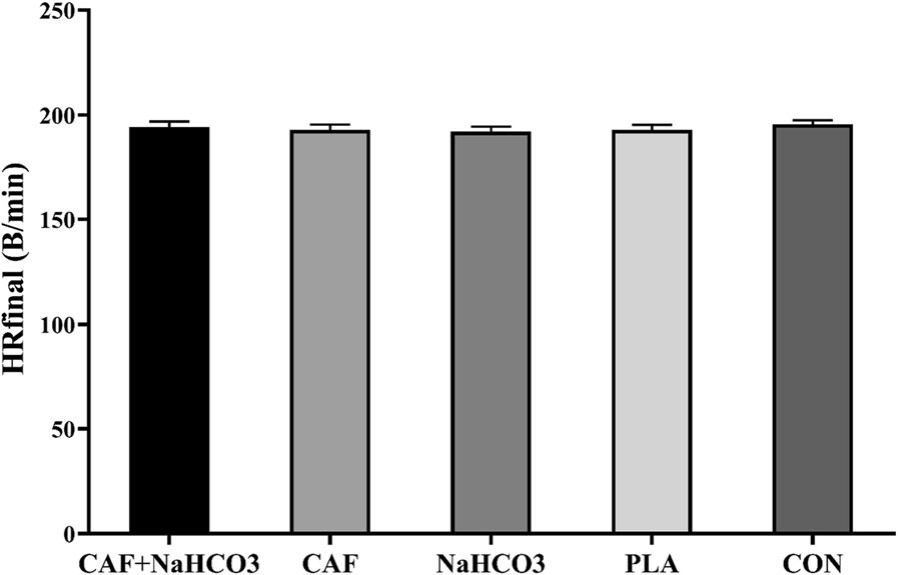
The maximum heart rate (beat/min) at the end of karate-specific aerobic test in each treatment. HR, heart rate; CAF, caffeine; NaHCO3, sodium bicarbonate; PLA, placebo; CON, control

The maximum HR at the end of KSAT, as demonstrated in Fig. 2., was not significantly different between conditions *F* [4, 28] = 1.112, *p* = 0.37, ${\eta}_P^2$ = 0.14. Moreover, as shown in Table 1. RPE was not significantly different between conditions at the completion of KSAT *F* [4, 28] = 2.051, *p* = 0.11, ${\eta}_P^2$ = 0.23. However, the RPE gradually increased from level two to level eight of KSAT in all conditions (*p* = 0.001).

**Table 1:** Rate of perceived exertion (RPE) in each level of the KSAT and the number of participants at each level. CAF, caffeine; NaHCO3, sodium bicarbonate; PLA, placebo; CON, control

Conditions		Level 2	Level 3	Level 4	Level 5	Level 6	Level 7	Level 8	Level 9
Caff+NaHCO3	RPE	6.63 ± 0.52	7.87 ± 1.81	9.25 ± 1.48	11.38 ± 2.07	12.75 ± 2.05	15.37 ± 1.50	18.29 ± 0.95	19
	*n*	8	8	8	8	8	8	7	3
NaHCO3	RPE	6.88 ± 0.83	8.13 ± 1.12	9.50 ± 1.51	10.87 ± 1.35	14.25 ± 1.75	16.75 ± 1.16	19.12 ± 0.64	19
	*n*	8	8	8	8	8	8	8	1
Caffeine	RPE	6.75 ± 0.71	8.50 ± 1.19	10.12 ± 1.73	11.87 ± 2.53	14.12 ± 2.53	16.88 ± 1.64	18.29 ± 0.95	19
	*n*	8	8	8	8	8	8	7	1
PLA	RPE	7.0 ± 1.69	8.75 ± 2.05	10.37 ± 1.92	12.25 ± 2.66	14.75 ± 2.60	17.50 ± 1.93	18.80 ± 0.45	–
	*n*	8	8	8	8	8	8	5	0
CON	RPE	7.25 ± 1.28	10.75 ± 2.31	12.25 ± 1.48	15.37 ± 1.06	16.87 ± 1.36	18.50 ± 0.76	19.5 ± 0.58	–
	*n*	8	8	8	8	8	8	4	0

The lowest RPE was observed in the CAF + NaHCO_3_ in all levels of KSAT. The RPE was similar in CAF, NaHCO_3_, and PLA conditions. The highest RPE was experienced in the CON condition. The RPE in levels three to seven was significantly lower in CAF + NaHCO_3_ compared to CON (*p* < 0.05); and in levels four and five in CAF and NaHCO_3_ compared to CON (*p* < 0.05). The general response of RPE to KSAT was a two-unit increase in RPE for each level increase in KSAT in all conditions (*p* = 0.001).

### Blood lactate

**Fig. 3:**
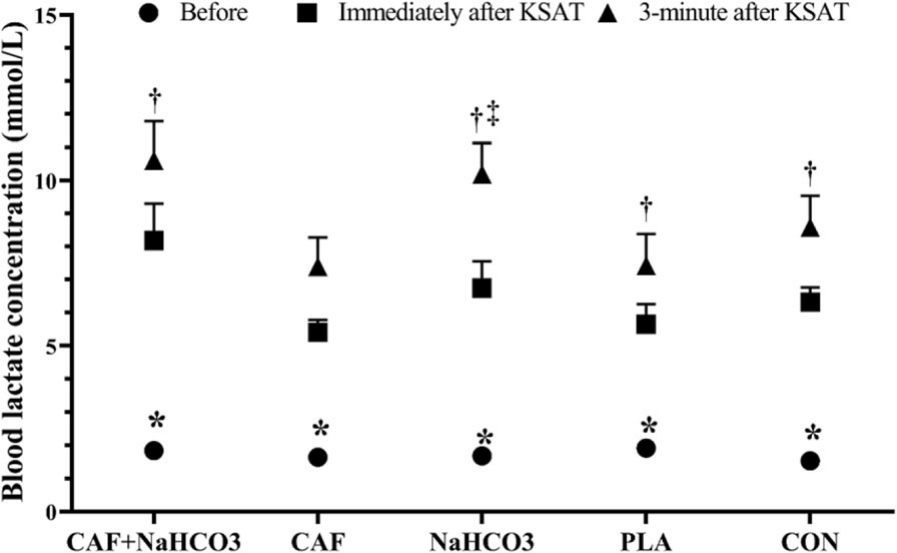
Blood lactate concentration (mean) before karate-specific aerobic test (KSAT), immediately after KSAT and 3 min after KSAT. CAF, caffeine; NaHCO3, sodium bicarbonate; PLA, placebo; CON, control. ^*^ Significant difference of Before KSAT with immediately after KSAT and 3-min after KSAT in the same condition (*P* < 0.05). ^**†**^ Significant difference between immediately after KSAT in the same condition (*P* < 0.05). ^**‡**^ Significant difference in 3-min after KSAT between NaHCO3 and CAF (*P* < 0.05)

The effects of different supplements *F* [4, 35] = 2.502, *p* = 0.06, ${\eta}_P^2$ = 0.22 and the interaction with time *F* (5.88, 51.46) = 2.105, *p* = 0.07, ${\eta}_P^2$ = 0.19 on BL were not significant. However, there was a significant time effect on BL *F* (1.47, 51.46) = 214.227, *p* < 0.001, ${\eta}_P^2$ = 0.86. BL increased significantly from baseline (1.73 ± 0.05) to immediately after KSAT (6.47 ± 0.49, *p* < 0.01) and 3 min after KSAT (8.845 ± 0.78, *P* < 0.01) in all conditions (Fig. 3). Furthermore, the lactate level was significantly higher at 3 min after KSAT than immediately after KSAT in CAF + NaHCO_3_ (*p* = 0.017), NaHCO_3_ (*p* < 0.01), PLA (*p* = 0.040) and CON (*p* = 0.018), but not in CAF condition (*p* > 0.05). The analysis of baseline data did not show any significant difference between conditions (*p* > 0.05).

Furthermore, the concentration of BL 3 min after KSAT was the highest in CAF + NaHCO3 and NaHCO3 and lowest in CAF and PLA (*p* = 0.003).

Pairwise comparisons revealed that the BL levels at 3 min following KSAT in CAF condition were significantly lower than NaHCO3 (*p* = 0.016); however, other pairwise comparisons did not show significant differences.

### Fatigue and gastrointestinal symptoms

Vertical jumps *F* [4, 28] = 1.86, *p* = 0.15, ${\eta}_P^2$ = 0.21 were not significantly different before KSAT in different conditions. Also, no participant reported severe abdominal discomfort throughout the study, and the total Hooper score was similar between conditions before KSAT.

## Discussion

This study examined the ergogenic effects of CAF and NaHCO_3_ when consumed together or separately on TTE, RPE, and BL during a Karate-specific aerobic test. We hypothesized that co-ingestion of CAF and NaHCO_3_ would have greater effect on athletic performance than CAF and NaHCO_3_ alone. However, the findings rejected the primary hypothesis and showed no additional benefits of co-ingesting CAF and NaHCO_3_ compared to CAF or NaHCO_3_.

Time to exhaustion in this study was defined as the duration of sustained high-intensity punches and kicks in KSAT until voluntary exhaustion. Our results indicated that all treatments increased TTE compared to PLA treatment during KSAT. Although the differences between treatments were not statistically significant, a close review of results showed a greater improvement in TTE in CAF+ NaHCO_3_ (9.3%) than CAF (5.8%) or NaHCO_3_ (8.9%) when compared to PLA. This result is consistent with previous studies using repeated sprint tests [36–38], Special Judo Fitness Test [25, 39], live Boxing [40] and simulated Taekwondo Combat [14]. In addition, Lopes-Silva et al. (2018), showed that ingesting NaHCO_3_ (0.3 g/kg BW) increased the attack time and improved performance during simulated Taekwondo combat [14]. Felippe et al. (2016), and Artioli et al. (2007) also demonstrated an improvement in the number of throws as a surrogate of Judo performance during a high-intensity intermittent special Judo fitness test after consuming 0.3 g/kg BW NaHCO_3_ [25, 39].

On the other hand, some evidence reported no improvements in continuous endurance exercise [41, 42], 4-km time trial [43], constant supramaximal exercise [44], 3-min all-out cycling test [45], and Judo combat [46] following ingestion of NaHCO_3_. These inconsistencies in the results could be due to differences in exercise protocols since multiple-bout high-intensity exercises appear susceptible to improvements with the ingestion of NaHCO_3_ [16, 47].

There is evidence to suggest a high level of intra-individual variability in the time to alkalotic peak following acute ingestion of NaHCO_3_ [47, 48]. Similarly, our participants showed large variability in TTE improvement (1.2–18.2%) after consumption of NaHCO_3_. The reason for such a large variation in TTE improvement after consumption of NaHCO_3_ is not clear. However, the existing data suggest that the absorption rate of bicarbonate (e.g. time to peak blood and pH) may differ greatly among individuals (10–85 min) [48, 49] which might explain the variability of performance among our participants.

TTE improvement in response to CAF treatment showed a smaller variability among our participants (2.5–7.3%). However, the overall TTE improvement in response to CAF treatment was lower than that of NaHCO_3_ treatment. One possible reason could be the duration of KSAT in this study. The duration of KSAT was approximately 10–12 min and the concentration of H^+^ ion was likely to be very high towards the end of KSAT. This exercise-induced acidosis would likely impair muscle contractions. Neutralizing the H^+^ by consumption of NaHCO_3_ appeared to be a responsible mechanism for TTE improvement. CAF supplementation, on the other hand, would have a different mechanism of action by delaying the pain and CNS fatigue. Previous research showed that CAF is more effective in long duration exercises, where CNS fatigue is more predominant [24].

The KSAT was developed to mimic the physiological demands of Kumite [29]. During this test, Karatekas completed high-intensity actions in 3 s while the active recovery time between bouts progressively decreased every 3 min. Therefore, at higher levels and near exhaustion, the contribution of anaerobic glycolysis was higher than other energy systems. The results of this study showed a greater improvement in TTE following NaHCO_3_ and co-ingestion compared to CAF alone. Interestingly, this improvement was accompanied by a higher BL accumulation immediately after and 3 min following KSAT upon NaHCO_3_ and co-ingestion conditions compared to the CAF condition. These changes are in line with previous studies on Special Judo Fitness Test [25, 39], simulated Taekwondo [14] and repeated sprint exercise in active females [36] after consuming 0.3 g/kg BW NaHCO_3_.

Despite an increase in extracellular bicarbonate, the sarcolemma has shown to be impermeable to bicarbonate. Therefore, it has been hypothesized that excess bicarbonate in the blood results in a greater efflux of H^+^ and lactate from the working muscle to the extracellular fluid [50]. In blood, excess H^+^ is buffered, by a mechanism in which there is a higher H^+^ gradient between the intra and extracellular spaces due to H^+^ buffering in blood. This will lead to a reduction in muscle acidosis and consequently, the inhibitory effect of acidosis on key enzymes of the glycolytic system such as glycogen phosphorylase and phosphofructokinase [51]. We speculate that ingestion of NaHCO_3_ alone or combined with CAF might have upregulated glycolysis pathway in karatekas during KSAT.

It is noticeable that our results showed an improvement in TTE following CAF ingestion while there was no difference in the lactate production rate in CAF compared to the PLA condition. Our results were consistent with studies showing an improvement in performance with no changes in BL following 5–6 mg/kg BW CAF supplementation [52–54]. We assumed that CAF ingestion could have a non-metabolic effect on KSAT. We did not observe any significant difference in RPE following CAF consumption compared to PLA or CON. However, participants could reach a higher level of KSAT upon CAF supplementation in comparison with PLA or CON. As a result, the CAF treatment might have assisted karatekas performing longer high-intensity actions during KSAT by affecting CNS mediated by adenosine receptor antagonists which can improve alertness and mood [24]. Another possible explanation for increased performance following CAF ingestion is the enhancement in the reuptake of K^+^ by activated muscle fibers [55].

Acute consumption of NaHCO_3_ has shown to be associated with GI discomfort [41]. To minimize the GI discomfort, a gradual loading strategy was adopted 3 days before a KSAT by splitting the daily bicarbonate dose into three equal portions consumed with breakfast, lunch, and dinner. The results of a study by McNaughton et al. (2001) suggested that increased blood carbonate levels following this loading strategy may be maintained 1 day after 0.5 g/kg BW of NaHCO_3_ consumption [30]. The result of this study confirmed that the loading strategy has a sustained effect. Besides, our participants did not report any GI discomfort during the protocol which was in agreement with previous studies conducted by Delextrat et al. (2018) on female basketball players [56], Driller et al. (2012) on well-trained cyclists [57], and Durkalec-Michalski et al. (2018) on CrossFit competitors [58].

We did not observe any differences in RPE between five conditions. Yet, the RPE score was lower in levels 6–8 after CAF+ NaHCO_3_ compared to other conditions. The exact mechanism of action behind this finding is unclear. However, several possibilities have been proposed that supports this finding [24]. Caffeine, as an adenosine receptors antagonist, can influence the central nervous system to sustain effort during exercise by reducing fatigue symptoms [59]. On the other side, peripheral changes such as extracellular accumulation of H^+^ might be modulated by III/IV muscle afferent feedback that stimulates some areas in the brain related to pain perception [60].

It is important to acknowledge the limitations of the current study. The speed and forces of attacks during KSAT was subjectively evaluated. Using accelerometer with wireless sensors attached to Karate gloves and force sensors attached in punching bag could have provided quantitative measures of the punching acceleration and the impact of both punches and kicks. Also, another limitation of this study was a small sample size. For consistency of training programs throughout the study, participants were recruited from only one Karate club, the largest karate club with most competitive karatekas. Further studies with a larger sample size may be needed to verify the findings of the present study.

In addition, the current study measured performance during a simulated test not real Karate combats. Chaabene et al. (2014) reported significant differences in physiological responses between official and stimulated Karate combats [4]. Thus, future research should investigate the ergogenic effects of supplements in competitions.

## Conclusion

Our results indicated that administration of NaHCO_3_ and CAF separately or combination could improve performance during a Karate specific aerobic test. Therefore, Karatekas may benefit from the consumption of CAF, NaHCO_3_, or co-ingestion of both prior to a Karate competition.

## Data Availability

Data and publication materials are available from the corresponding author on reasonable request.

## Abbreviations

ANOVA: Analysis of variance
BL: Blood lactate
CAF: Caffeine
CMJ: Countermovement jump
CNS: Central nervous system
CON: Control
ES: Effect size
KSAT: Karate-specific aerobic test
NaHCO_3_: Sodium bicarbonate
PLA: Placebo
RPE: Perceived exertion
SD: Standard deviation
TTE: Time to exhaustion
